# Polymer nanodiscs support the functional extraction of an artificial transmembrane cytochrome

**DOI:** 10.1016/j.bbamem.2024.184392

**Published:** 2024-10-15

**Authors:** Benjamin J. Hardy, Holly C. Ford, May Rudin, J.L. Ross Anderson, Paul Curnow

**Affiliations:** School of Biochemistry, University of Bristol, UK

**Keywords:** Membrane protein purification, Polymer nanodisc, Protein design, Cytochrome

## Abstract

Polymer nanodiscs are an attractive alternative to surfactants for studying integral membrane proteins within their native lipid environment. Here, we investigate the use of such polymers to isolate a computationally-designed *de novo* membrane cytochrome named CytbX. We show that the block copolymers known as CyclA-Pols can efficiently extract CytbX directly from biomembranes and are compatible with the downstream purification and biophysical characterisation of this artificial protein. CyclAPol-solubilised CytbX is well-folded and highly robust with properties that are essentially identical to those observed for the same protein in a detergent micelle. However, electron transfer to CytbX from a diffusive flavoprotein is substantially faster in micelles than in the nanodisc system. Our results confirm that polymer nanodiscs will be a useful tool for the ongoing study and application of *de novo* membrane proteins.

## 1 Introduction

**Fig. 1:**
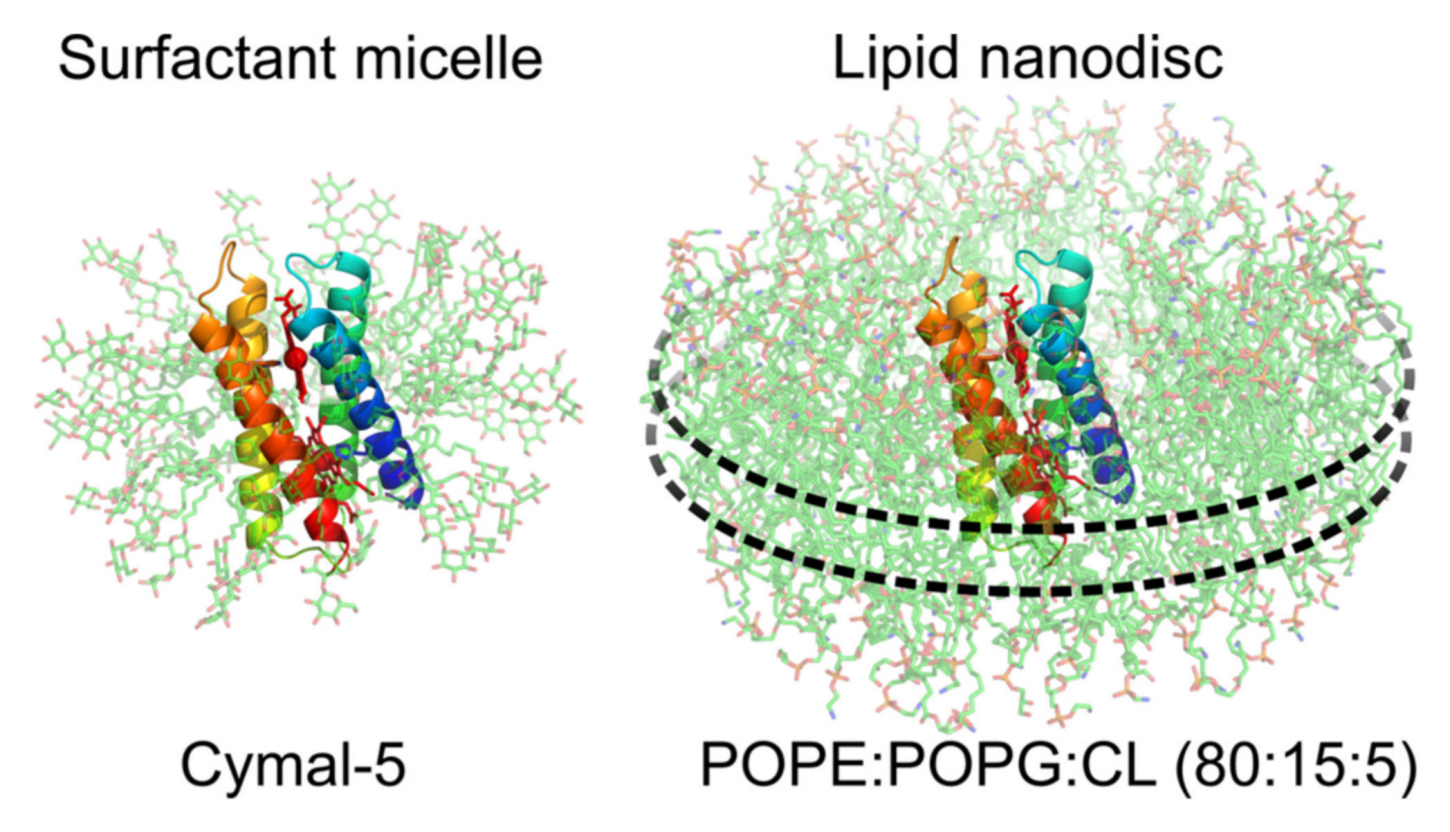
Models of the *de novo* cytochrome CytbX embedded in a surfactant micelle and a lipid nanodisc. Protein backbone is shown coloured as rainbow, heme cofactors coloured red. The nanodisc composition is chosen to mimic the *E. coli* inner membrane and the approximate position of the polymer belt surrounding the nanodisc is indicated by dashed lines. Models generated with CHARMM-GUI [18] and visualised in PyMOL. (For interpretation of the references to colour in this figure legend, the reader is referred to the web version of this article.)

The characterisation of integral membrane proteins *in vitro* often requires their isolation from cell membranes in solubilising surfactant (*aka* detergent) micelles [1]. However, this extraction step can be a significant experimental bottleneck because the solubilising surfactant must be carefully matched to the specific protein-of-interest in order to avoid non-specific aggregation or protein mis-folding [2]. Overcoming this bottleneck often requires the resource-intensive screening of multiple detergents [3,4]. Additionally, membrane proteins can be delipidated in the protein-detergent complex and in some cases this disruption to the natural lipid environment is associated with a loss of stability and function [5]. These issues have motivated the search for surfactant alternatives such as Amphipols [6] and peptide-based nanodiscs [7,8] which act as generic solubilising agents and do not need to be matched to the specific properties of the target protein (Fig. 1). Amphipathic polymers such as styrene-maleic acid [9], styrene-acrylic acid [10], diisobutylene-maleic acid [11] and cycloalkane-substituted polyacrylate [12] can extract integral membrane proteins along with their local lipids directly from the cell membrane *via* a ‘cookie-cutter’ process [13,14]. These various solubilising polymers thus support the isolation of the protein within a biologically-relevant lipid sheath – dubbed a *memtein* [15].

We recently described the computational design and cellular production of an artificial transmembrane diheme cytochrome that we named CytbX [16]. This *de novo* protein is a four-helix bundle that non-covalently binds two molecules of *b*-type heme at the bundle core. The designed protein sequence of CytbX was genetically-encoded for recombinant expression in *Escherichia* (*E*.) *coli*. We demonstrated that CytbX could be efficiently extracted directly from recombinant cell membranes in the nonionic detergent 5-cyclohexyl-1-pentyl-β-D-maltoside (Cymal-5) and that this stable protein-detergent complex could be purified by affinity chromatography using a His_10_ tag [16]. Detergent-purified CytbX contained two non-covalent *b*-type hemes, apparently recruited from the endogenous heme pool during protein biosynthesis. The two hemes exhibited split redox potentials of −14 and −120 mV, and this splitting was subsequently found to depend upon the low dielectric of the micelle interior, which amplified electrostatic interactions between the heme groups [17]. Purified detergent-solubilised CytbX could engage in rapid inter-protein electron transfer with a promiscuous electron donor, the *E. coli* flavoprotein FLDR [16]. This work is thus a significant step toward the use of artificial membrane proteins in cellular bioenergetic and redox reactions. However, we have not yet assessed the activity of CytbX in authentic lipid bilayers.

Here, we use amphipathic polymers to extract CytbX directly from cell membranes in a lipid nanodisc. Broadly, we seek to determine to what extent the observed properties of CytbX depend upon the solubilisation conditions. Is CytbX folded within the membrane nanodisc? Are the two CytbX hemes retained in these preparations? Are the heme redox potentials consistent across different solubilisation systems? Can lipid nanodiscs support protein-protein electron transfer reactions? Our results show that CytbX is compatible with direct extraction into various lipid nanodiscs, with CyclAPol copolymers demonstrating particularly high solubilisation efficiency. All of the key properties of CytbX, including cofactor loading and redox potential, are retained in these CyclAPol particles. The exception to this is protein-protein electron transfer, which is slower in the nanodisc preparation. These data will support the further study of CytbX and other *de novo* membrane proteins, as well as the application of these proteins in synthetic biology.

## 2 Materials and methods

### 2.1 Polymer screening

The CytbX-GFP fusion protein containing a His10 affinity tag was cloned into plasmid pET28 for recombinant production as previously described [16]. The host strain was *Escherichia coli* strain C43, a derivative of BL21(DE3) selected for improved production of recombinant integral membrane proteins [19]. Following induction with 0.1 mM IPTG, cells were washed in 1 × PBS and lysed in a continuous-flow disruptor (Constant Systems Ltd) at 25 KPSI. The lysate was centrifuged at 10,000 x *g* for 15 min to remove unbroken cells. Cellular membranes were obtained by centrifuging the clarified lysate at 180,000 x *g* for 1 h. Pelleted membranes were resuspended in *Membrane Buffer* (50 mM sodium phosphate, 150 mM NaCl and 5 % glycerol at pH 7.4), adjusted to 40 mg/ml by wet weight per volume, and homogenised in a hand-held glass homogeniser. The membrane suspension was divided into 1 ml aliquots before adding 25 mg each of individual synthetic polymers from a commercial screening kit (Cube Biotech #18295). After 3 h at room temperature the reactions were centrifuged at 12,000 x *g* for 10 mins. The soluble fraction was removed and applied to 50 μl Ni-IDA resin slurry (Generon) washed in Membrane Buffer. After overnight incubation, the resin was pelleted at 3000 x *g* for 30 s. The unbound protein was removed and the resin was washed once in Membrane Buffer. Bound protein was eluted from the resin by incubating with 500 μl Membrane Buffer containing 0.25 M or 0.5 M imidazole. The relative concentration of nanodisc-solubilised, binding-competent CytbX-GFP within the eluate was determined by fluorescence spectroscopy of the GFP using excitation at 490 nm and measuring emission at the approximate maximum of 512 nm.

### 2.2 Protein purification

Large-scale protein production made use of two recombinant CytbX constructs. In the first instance, CyclAPol-solubilised CytbX-His was purified on a 1 ml Ni-NTA column essentially according to published protocols described for the detergent Cymal-5 [16]. Subsequently, a second expression construct was used in which CytbX was fused to a thrombin-cleavable triplet StrepII tag. This CytbX-Strep construct was cloned into pET29 and expressed in *E. coli* C43 cells. Cells were grown to an optical density of 0.9 in LB broth before induction with 0.1 mM IPTG; at the point of induction the growth media was supplemented with 25 mg/l aminolevulinic acid to support heme synthesis. After post-induction growth at 37 °C for 2 h, cell lysis and membrane isolation were performed as described above. 10 ml of cell membranes were treated with 2.5 % *w/v* CyclAPol C8-C0–50, sold commercially under the name Ultrasolute™ Amphipol-18 (Cube Biotech #18321) and here called CyclAPol for brevity. Insoluble material was removed by centrifugation at 180,000 x *g*. The supernatant, containing soluble membrane nanodiscs, was either incubated overnight with 5 ml Strep-tactin slurry or applied to a 5 ml Strep-tactin column (IBA Life Sciences). In some instances 0.2 M arginine was introduced to the column buffer for charge screening but this had no obvious influence on column binding or sub-sequent nanodisc behaviour. After washing, column-bound polymer nanodiscs were eluted with 2.5 mM desthiobiotin.

### 2.3 Protein characterisation

UV–Vis spectroscopy was performed with a Cary 60 instrument. The concentration of CytbX-Strep was determined using the oxidised heme absorbance band at 417 nm using a calculated heme extinction coefficient of 155,200 M^−1^.cm^−1^ [16]. The calculated ε_280_ for CytbX-Strep is 23,490 M^−1^.cm^−1^ meaning that the 280 nm absorbance for this protein is relatively low, and is impacted by heme absorbance and light scattering. Thus the heme signal is used for determining concentration, assuming 100 % heme loading. Absorption measurements were made in a 1 ml quartz cuvette. The spectrum of purified CytbX, containing oxidised heme, was recorded first. To obtain a reduced spectrum of the same sample, the protein heme was reduced *in situ* with sodium dithionite. A few grains of dithionite powder were drawn into a disposable plastic tip attached to a P200 pipette. The pipette tip was transferred to the cuvette and dithionite introduced to the sample by gentle pipetting. Reduced spectra were recorded immediately but were not found to significantly change after 5 min incubation. SDS-PAGE used a 4–20 % gradient Tris-Glycine gel (NuSep) and Coomassie staining. For circular dichroism of CytbX-Strep the protein was adjusted to 0.1 mg/ml (*w/v*) in Membrane Buffer and data collected in a 1 mm pathlength cuvette in a Jasco J-1500 instrument. Subsequent calculations were based on the mean residue weight of CytbX-Strep only. Size exclusion chromatography in Membrane Buffer used a 10/300 Superdex 200 column (Cytiva) at a flow rate of 0.5 ml/min. Dynamic Light Scattering was performed using a Malvern Panalytical Zetasizer Nano ZSP instrument in Membrane Buffer without glycerol at protein concentrations of 0.125–0.5 mg/ml at 25 °C. Count rates were at least 150 kcps over 50s total. Redox potentials were determined by optically-transparent thin-layer electrochemistry as described [16].

### 2.4 Electron transport assays

The *Escherichia coli* NADPH-dependent flavodoxin reductase (UNIPROT P28861; PDB 1FDR; here called FLDR *as per* McIver [20] but also termed Fpr in the literature [21]) was purified after recombinant expression. This utilised an existing pBAD construct containing FLDR with a C-terminal His-tag. FLDR was overexpressed in *E. coli* T7 Express cells using 0.1 % *w/v* arabinose, and purified from the resulting cell lysate on Ni-NTA resin by standard affinity chromatography methods. Electron transport assays were performed under anaerobic conditions in a glove box as described [16]. 2 μM CytbX, as determined from heme absorbance, was incubated with 5 μM FLDR for 1 min prior to the addition of 100 μM NADPH. UV–Vis spectra were captured between 183 and 911 nm at 1.5 s intervals using an Ocean Optics DH-2000 light source and an Ocean Optics SR miniature spectrophotometer. The integration time was 5 ms with averaging of 100 scans at boxcar width 2. The extent of heme reduction at each timepoint was determined from the Soret band, using wavelengths of 416 nm and 428 nm for the oxidised and reduced heme respectively.

### 2.5 Electron microscopy

A carbon film 300 mesh copper grid (Electron Microscopy Sciences) was prepared by glow-discharge at 40 mA for 30 s on a GloQube Plus (Quorum Technologies). CytbX nanodiscs were diluted to 5 μM hemoprotein in Membrane Buffer and 5 μl was applied to the grid. After 1 min, unadhered sample was removed by blotting. The grid was stained three times with 3 % (*w/v*) uranyl acetate with excess liquid removed by blotting after each step. Images were acquired on a Technai 12 transmission electron microscope operating at 120 kV with a Ceta 16 M camera (ThermoFisher Scientific). Single-particle image processing was performed using EMAN2.91 [22]. A total of 285 micrographs, with a pixel size of 3.27 Å, were taken forward for particle picking after evaluation with “*e2evalimage.py*”. Automated CTF processing was performed using “*e2ctf_auto.py*”. Automated particle picking was performed using the NeuralNet feature of “*e2boxer.py*” [23]. To train the neural network, examples of the small 15–25 nm particles were given as ‘good’ particles and examples of the larger 50–100 nm particles were given as ‘bad’ particles, in additional to background references. Bispectrum-based class averaging was used to generate 56 class averages (over 4 iterations) from 28,354 particles.

### 2.6 Computational model-building

Computational models of monomeric CytbX embedded in a POPE/POPG/CL membrane nanodisc and a Cymal-5 micelle were generated using Membrane Builder in CHARMM-GUI [18]. CytbX was pre-oriented with PPM3.0 (http://opm.phar.umich.edu/ppm_server) [24]. Since polymers were not available, approximated nanodiscs were built using the parameters for MSP1. For micelle-building with Cymal-5 (BCY5M) best results were from setting the radius to 15 Å with an aggregation number of 60, slightly higher than the experimental aggregation number of ~47 (https://www.anatrace.com).

## 3 Results

### 3.1 Polymer screening

**Fig. 2:**
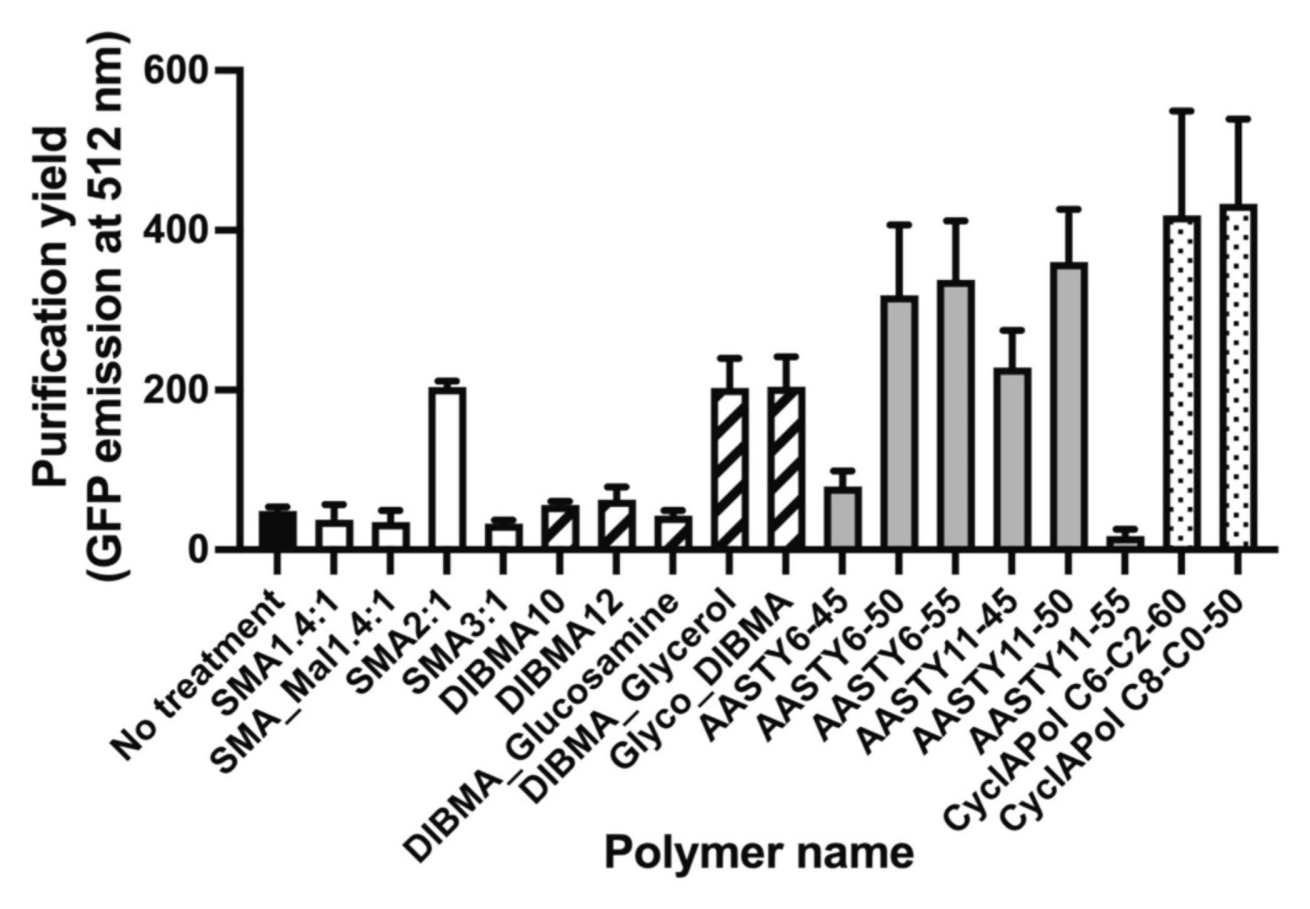
Screening polymer nanoparticles for the successful purification of Histagged CytbX-GFP. Cell membranes were treated with each of the polymers shown. Solubilised membranes were applied to an Ni-IDA slurry and nanodisc-soluble, resin-binding CytbX-GFP was eluted with imidazole and quantified by fluorescence. Data are mean ± range from duplicate experiments. *SMA*, styrene-maleic acid; *DIBMA*, diisobutylene-maleic acid; *AASTY*, styrene-acrylic acid; *CyclAPol*, cycloalkane-substituted polyacrylate.

A fluorescence-based screen was used to determine whether various polymer formulations could support the efficient membrane extraction and subsequent purification of CytbX in nanodiscs. CytbX-GFP with a C-terminal His-tag was expressed in recombinant *E. coli*, where it is known to be targeted to cellular membranes [16]. Recombinant cell membranes were isolated by centrifugation and treated with nanodisc-forming polymers, and the GFP signal was used to assess the extent of membrane solubilisation and the binding of nanodisc-solubilised CytbX-GFP to a Ni^2+^ affinity resin. The highest purification yields from this screen were observed in the CyclAPols, SMA2:1, Glyco-DIBMA, DIBMA-glycerol, and some of the AASTYs (Fig. 2). The polymer giving the greatest purification yield, CyclAPol C8-C0–50, is available commercially under the brand name Ultrasolute Amphipol 18. The CyclAPols were developed for enhanced membrane solubilisation while retaining the native lipid profile in the nanodisc particle [12,13] and are compatible with structural biology [25]. CyclAPol C8-C0–50 was therefore selected for all further experiments described below, and hereafter is referred to as CyclAPol for simplicity.

### 3.2 Protein purification

CytbX was purified from large-scale expression cultures using either a previously-described His-tag method [16] or using a serial triplet StrepII tag. This latter construct, which we call CytbX-Strep, featured three consecutive StrepII sequences (WSHPQFEK) separated by flexible linkers. The full sequence of the construct is provided as supplementary material. We found in preliminary work that CytbX-Strep was expressed by recombinant cells at much higher levels in comparison to the Histagged construct, and so CytbX-Strep was considered to be a better target for high-yield purification in the CyclAPols.

**Fig. 3:**
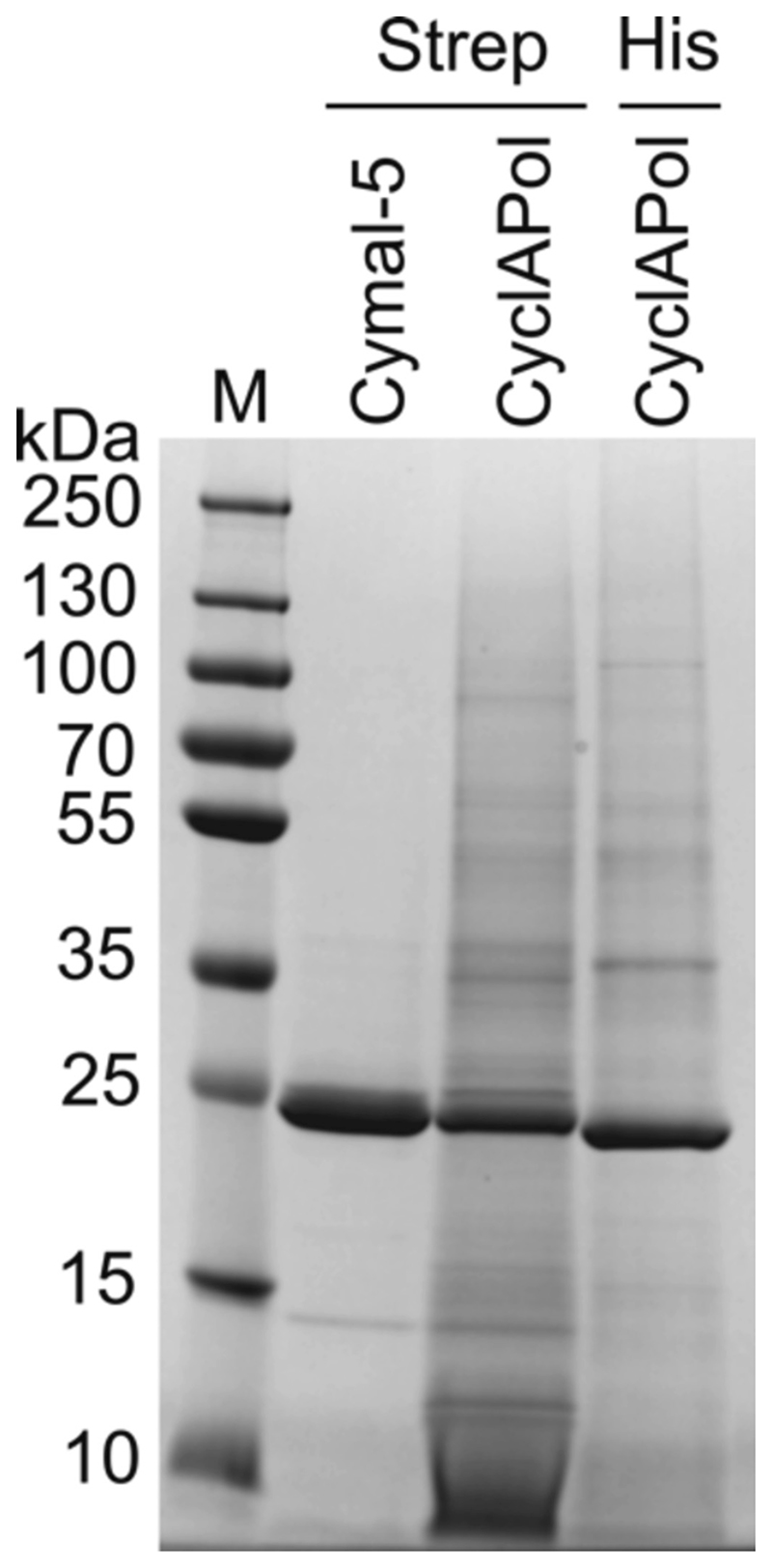
Affinity purification of the *de novo* membrane protein CytbX using different solubilisation reagents and tag systems. CytbX with a triplet StrepII tag (*Strep*) is purified to homogeneity *via* a Streptactin affinity column in the surfactant Cymal-5, but the same construct is only semi-purified after protein extraction in the CyclAPol Amphipol-18. A similar result was seen upon purifying CytbX-His_10_ in CyclAPol nanodiscs (*His*). Theoretical molecular weights for the protein constructs are 18.9 kDa (*Strep_3_*) and 15.6 kDa (*His*); membrane proteins often migrate anomalously on SDS-PAGE and the theoretical weights approach the observed migration pattern once the Rath-Deber correction factor of 1.13 is applied [27].

As expected, membrane solubilisation with 2.5 % *w/v* CyclAPol C8-C0–50 at large scale replicated the success of the small-scale screen with almost all of the extracted protein remaining in solution after ultra-centrifugation. CyclAPol-solubilised CytbX-Strep was purified using a Strep-tactin column. Column binding was relatively weak, as sometimes seen for nanodisc proteins [26], meaning that protein recovery was poor and yields were 0.3 mg CytbX-Strep per litre bacterial culture. This was in contrast to data obtained with Cymal-5-solubilised CytbX-Strep, which bound very well to Strep-tactin and was recovered at 3 mg per litre culture. Analysis by SDS-PAGE determined that CytbX-Strep did constitute the major protein band after purification, but appeared to copurify with a non-specific set of other proteins (Fig. 3). This non-specific background differed between different purification tags, and is in contrast to the very pure preparations obtained in Cymal-5 (Fig. 3 and [16]).The semi-pure nanodisc preparations of CytbX-Strep were taken forward for further analysis. From this point onwards, only CytbX-Strep is used and so is simply referred to as “CytbX” for brevity.

### 3.3 Characterisation of protein-nanodisc particles

**Fig. 4:**
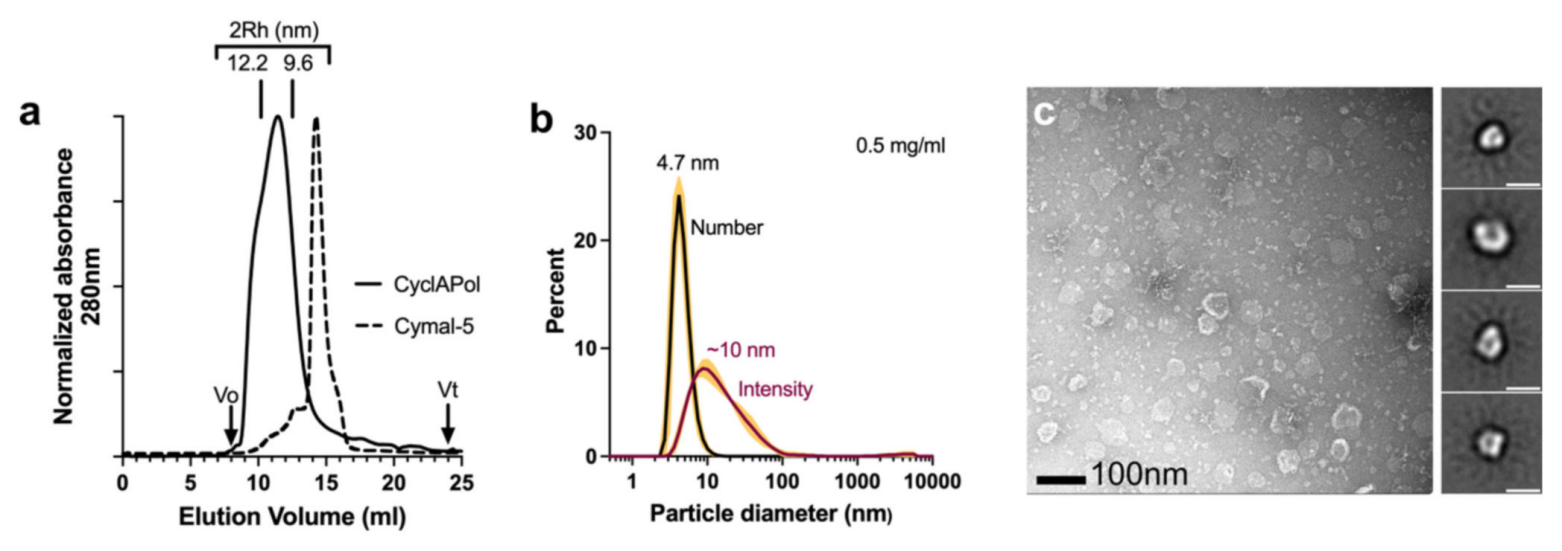
Size distribution of CytbX after purification in CyclAPol. (a) Size exclusion chromatography of CytbX in CyclAPol nanodiscs (solid line) shows a broad distribution with a distinct shoulder compared with the same protein in Cymal-5 micelles (dashed line). Elution volumes of standards with known hydrodynamic diameter (2Rh) shown. (b) Dynamic light scattering of CytbX in nanodiscs indicates a distribution of smaller particles (dominating population distribution by *Number*) and larger particles (dominating *Intensity*). Data are mean ± SD from 3 technical repeats on the same sample, error bars in yellow. Protein at 0.5 mg/ml. (c) Negative stain TEM confirms the presence of numerous irregular smaller particles interspersed with a few larger aggregates. Right panels are representative 2D class averages of the smaller particles, scale bar 20 nm. Data in (b) and (c) were recorded on samples recovered after SEC. (For interpretation of the references to colour in this figure legend, the reader is referred to the web version of this article.)

Fig. 4 shows the results of using size exclusion chromatography (SEC), dynamic light scattering (DLS) and Transmission Electron Microscopy (TEM) to assess the approximate size and dispersity of CyclAPol-solubilised CytbX. Overall, the nanodisc preparations were more heterogenous than detergent preparations. In SEC (Fig. 4a) the bulk of the particles eluted at a position equivalent to ~10 nm hydrodynamic diameter (2Rh). This was largely confirmed by DLS (Fig. 4b and Supplementary Fig. S1), although this analysis was complicated by the sample heterogeneity and converting the raw intensity data to particle number showed a shift to smaller apparent size. The sample heterogeneity was also evident in negative-stain TEM (Fig. 4c) which showed a mixture of small particles and large aggregated particles. Given that these larger particles are only evident in TEM they may represent grid deposition artifacts. Class averaging of the smaller particles determined them to be on the order of 10–20 nm diameter, consistent with other studies of CyclAPol nanodiscs [25]. The full set of class averages are provided as Supplementary Fig. S2. Further image processing was not pursued because of the small size of CytbX.

### 3.4 Protein characterisation

**Fig. 5:**
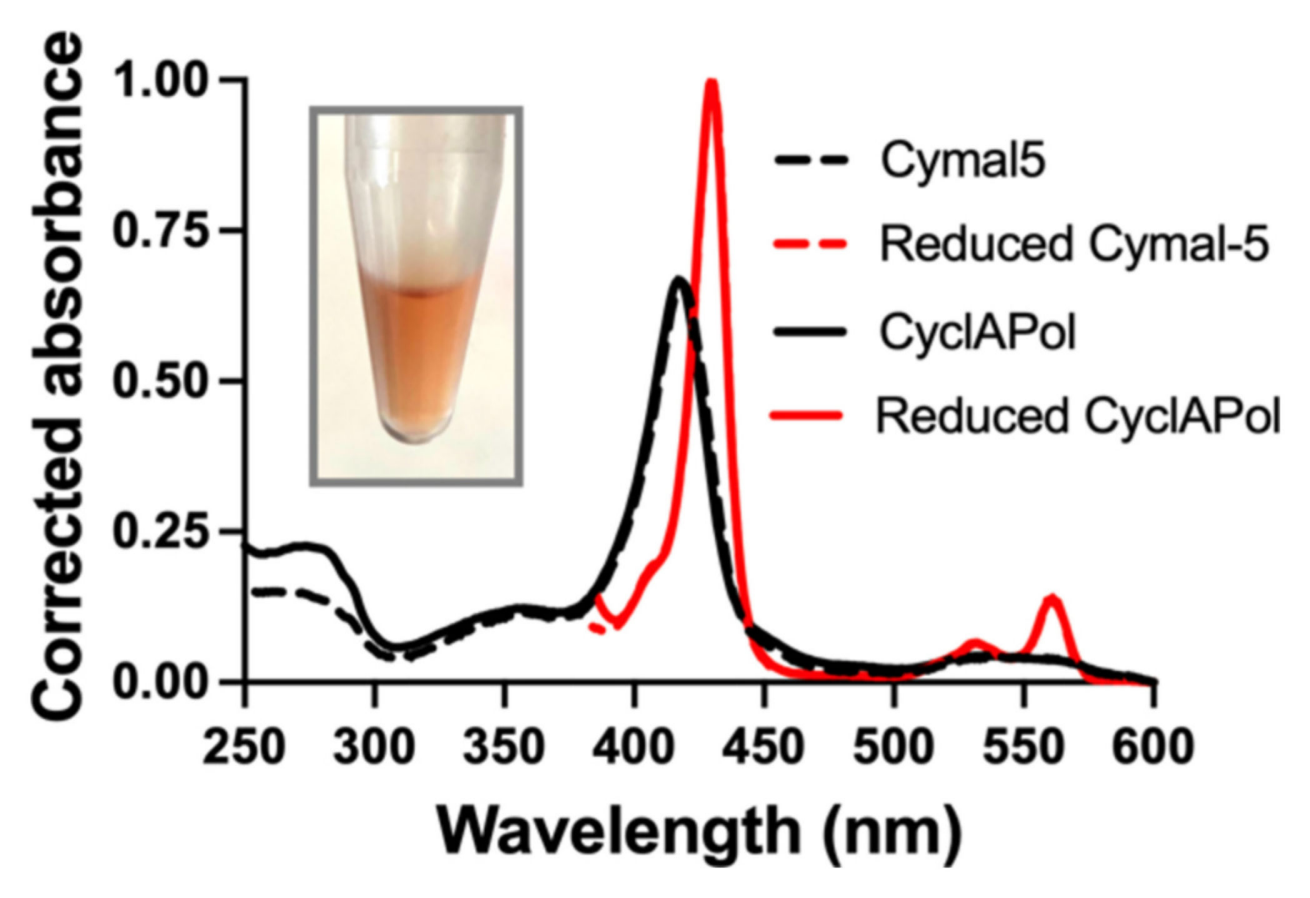
Spectroscopic characterisation of CyclAPol-solubilised CytbX. UV–Vis absorbance spectroscopy is consistent with nanodisc CytbX coordinating two *b*-type hemes, with the heme spectrum almost exactly juxtaposed with that of CytbX in Cymal-5. Inset photograph shows the expected red colour of nanodisc-purified CytbX. (For interpretation of the references to colour in this figure legend, the reader is referred to the web version of this article.)

Purified CytbX in nanodiscs was red in colour, consistent with the binding of endogenous heme. Absorption spectroscopy confirmed the presence of hexacoordinate *b*-type heme, and both oxidised and dithionite-reduced heme spectra for the nanodisc sample were indistinguishable from detergent-solubilised CytbX (Fig. 5a). The protein absorbance band at 280 nm was slightly higher for the amphipol sample, consistent with the observed co-purification of other non-heme proteins. The CyclAPol polymer does not strongly absorb within the UV, and so does not contribute to this signal.

### 3.5 Secondary structure

**Fig. 6:**
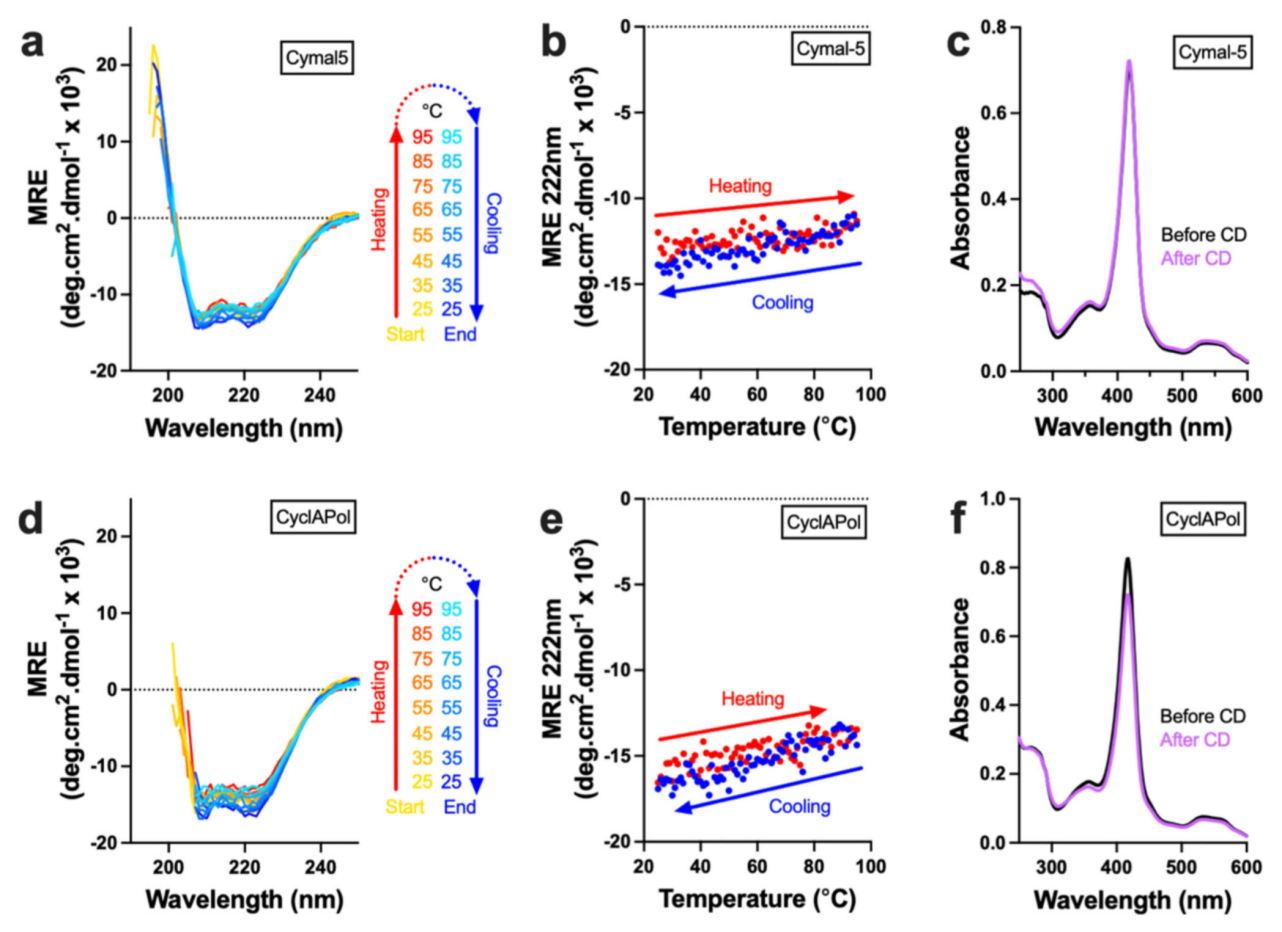
Secondary structure and thermostability of CytbX. (a, d) Circular dichroic spectra for CytbX in Cymal-5 and CyclAPol nanodiscs. (b, e) The CD signal is essentially preserved during reversible heating to 95 °C. The gradual and minor linear change in the signal upon heating is recovered when the sample is cooled. (c, f) Minimal change in the heme absorbance spectra collected before and after the thermal melts shown in panels a–d.

The secondary structure of nanodisc-solubilised CytbX was determined by circular dichroism, and is directly compared here to equivalent data in Cymal-5 (Fig. 6). The interpretation of these data is impacted by the presence of other proteins within the nanodisc, which will contribute to the CD signal, and the uncertainty in background correction for the nanodisc sample. Nonetheless, in both systems the purified protein was clearly alpha-helical, as expected for the CytbX design. This signal was only modestly affected by heating to 95°, suggesting that nanodisc-solubilised CytbX retains the thermostability previously observed in detergent micelles [16].

### 3.6 Redox potentiometry

**Fig. 7:**
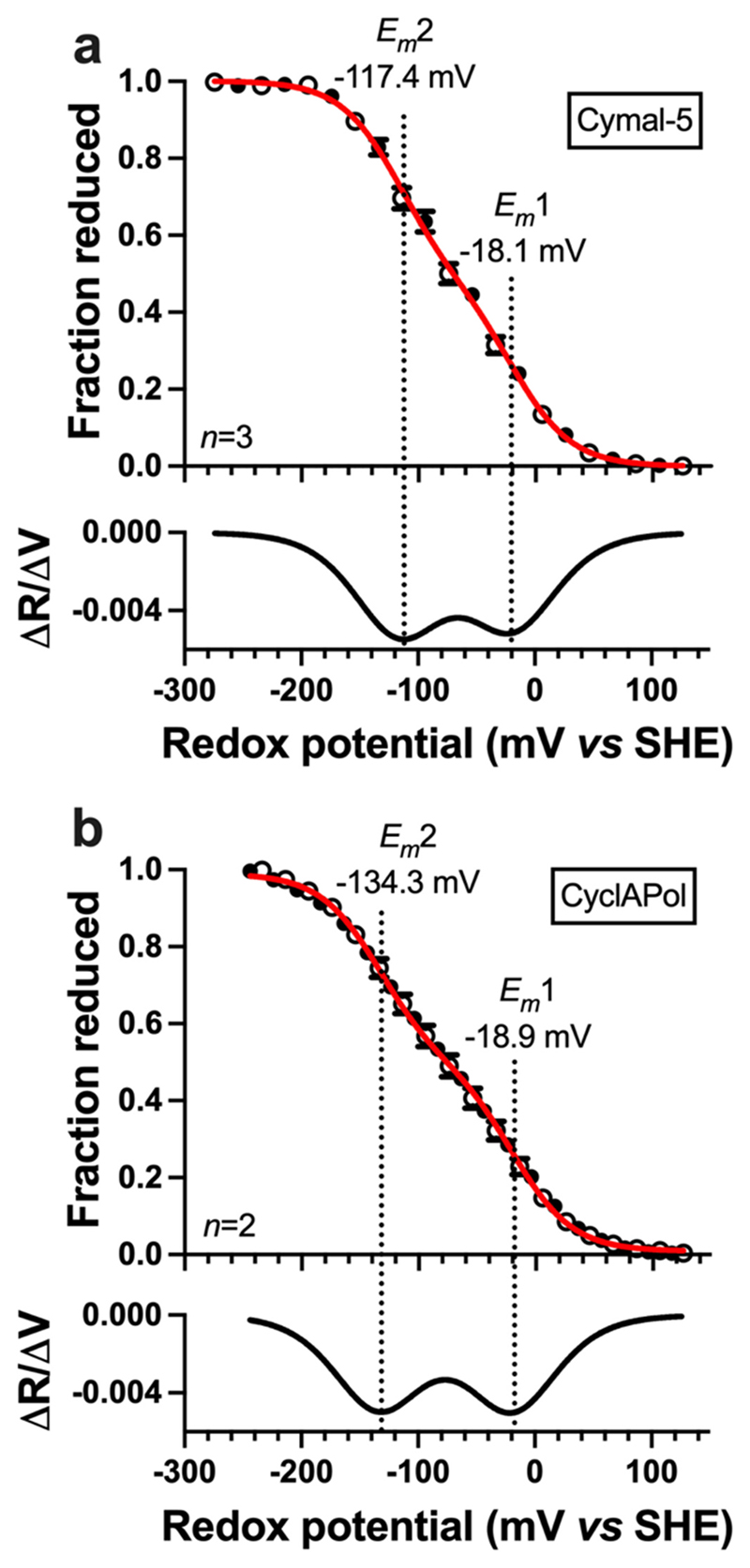
Redox potentiometry of Strep-tagged CytbX. (a) Redox measurements in surfactant micelles. (b) Redox measurements recorded in CyclAPol nanodiscs. Data are mean ± SD from 2 or 3 repeats as shown. *E_m_* values are from curve fitting to the 2-electron Nernst equation. The relevant first-derivative plots are shown underneath each graph.

The experimental redox potentials of diheme CytbX were determined *via* optical potentiometry (Fig. 7). The amphipol-solubilised protein showed split midpoint potentials at − 18.9 mV and − 134.3 mV. The value of the higher potential is the same as CytbX in Cymal-5, but the lower potential is slightly more negative by about − 17 mV. The observed redox potentials reflect the burial of CytbX within a hydrophobic environment, which enhances proximity-induced heme redox splitting [17]. The similarity of the micelle and nanodisc data suggest that this environmental effect is relatively similar between these two systems.

### 3.7 Electron transport

**Fig. 8:**
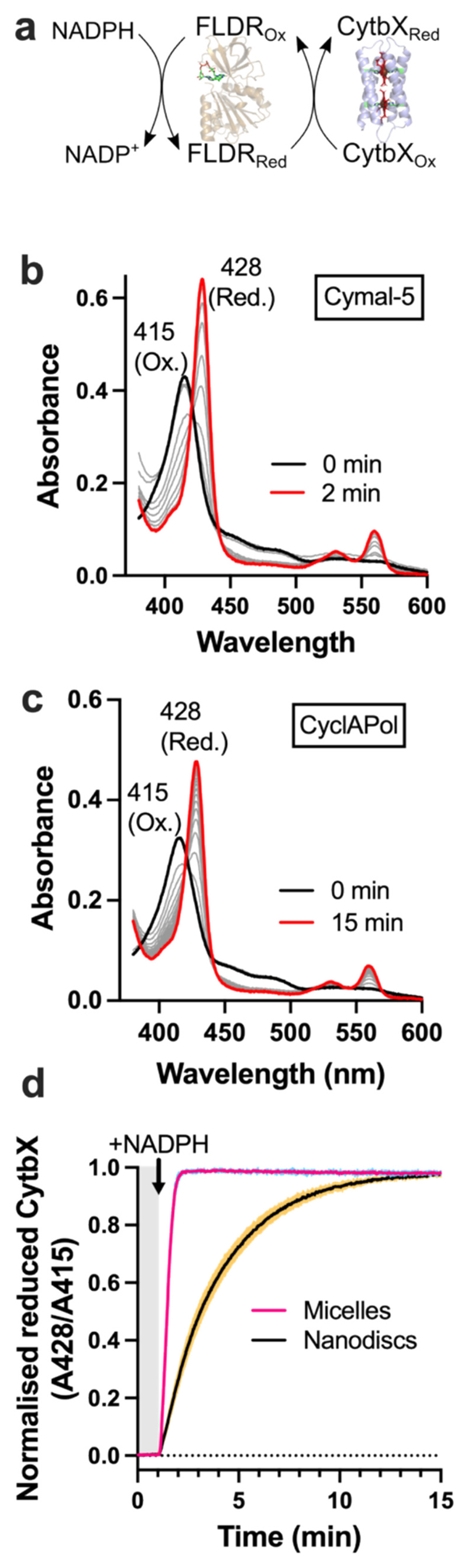
Electron transfer from *E. coli* FLDR to CytbX. (a) The *E. coli* NADPH-dependent flavodoxin reductase transfers electrons from NADPH to CytbX as shown. Ox, oxidised; Red, reduced. CytbX is fully reduced by the reaction in both (b) Cymal-5 micelles and (c) CyclAPol lipid nanodiscs. Both panels show initial scans taken at 0, 30 and 60 s, at which point NADPH is introduced to start the reaction. After that point, panel (b) shows scans collected every 5 s for the next minute; panel (c) shows scans taken every 30 s up to 10 min, and every minute thereafter to 15 min. These datapoints were chosen to try and show equivalent spectral features between the two reactions. (d) FLDR-mediated reduction of CytbX is slower in the nanodisc system. Data shown in panel (d) are mean ± SD, n = 3; for micelle experiments the error bounds shown in blue are similar to the line thickness. (For interpretation of the references to colour in this figure legend, the reader is referred to the web version of this article.)

We previously observed electron transfer to detergent-solubilised CytbX from the versatile soluble *E. coli* NADPH-Flavodoxin oxidoreductase (FLDR) [16,20,28]. Such protein-protein electron transfer events can be very different between detergent-solubilised and nanodisc-solubilised heme proteins (*e.g*. [29]). Nanodisc-solubilised CytbX was fully compatible with this same assay and was almost completely reduced by FLDR upon introduction of NADPH (Fig. 8:b–c). There was no substantial difference between the observed reaction rate between micelle-solubilised CytbX-Strep studied here and similar experiments reported earlier with CytbX-His [16]. However, FLDR-mediated reduction of CytbX in nanodiscs was markedly slower than in micelles, with an initial linear rate approximately 5-fold slower in the nanodisc system (Fig. 8b–d).

## 4 Discussion

This paper describes the first purification of the *de novo* membrane protein CytbX in polymer nanodiscs. This allows for the study of CytbX in a membrane-like environment, as opposed to the delipidated detergent micelles used previously [16]. While purification yields are notably lower in nanodisc preparations due to poor column binding, the results confirm that many of the observed properties of CytbX are largely independent of the particular solubilisation method that is used. This provides further support for a model in which CytbX is able to fold and form the hemoprotein complex within biological membranes [16].

Our results show that CytbX can be efficiently solubilised by CyclAPol polymers and subsequently purified using both streptactin and Ni-NTA affinity resins (Figs. 2 and 3). However, in both cases background contaminants are evident, presumably from non-specific binding or from the random capture of other membrane proteins within the nanodisc. More stringent solubilisation conditions may help to reduce this background signal, and success has been reported with as little as 0.1 % CyclAPol for membrane solubilisation [25].

Nanodisc-solubilised CytbX can be successfully imaged by single-particle electron microscopy (Fig. 4). This bodes well for further analysis at higher resolution using cryo-EM. A key advantage of lipid nanodiscs lies in their broad compatibility with cryogenic methods whereas detergents, especially high-CMC detergents like Cymal-5, can interfere with vitrification and image processing [30,31] and even cause structural artifacts [32]. While the CytbX protomer is too small for high-resolution EM, it should be possible to increase the molecular weight by designing fusion partners or exploiting binding interactions with other proteins [31]. This approach is of particular interest since we have found that CytbX does not readily crystallise in common membrane protein screens. Nanodiscs could also allow the reconstitution of CytbX in different bilayer formulations in order to explore lipid compatibility, lipid-protein interactions and protein folding in a way that is essentially impossible in detergent systems (*e.g*. [33]). Additionally, various nanodiscs have been shown to preserve membrane protein complexes, lipid contacts and cofactor interactions [34–36] so could be used to study whether CytbX can be redesigned to form functional assemblies with other membrane proteins, lipids, and small molecules. Membrane nanodiscs are compatible with bioelectronic devices [37,38] and can be used for biochemical and biophysical research that exploits surface immobilisation, solid-state NMR, or cell-free expression [8]. Our work here thus establishes the nanodisc approach as a versatile platform for the ongoing study and application of the *de novo* cytochrome CytbX.

We find here that there are only marginal differences in the properties of CytbX between the micelle and nanodisc systems tested (Figs. 5–7). The most notable distinction is the slower rate of reaction between CytbX and the water-soluble flavoprotein FLDR in nanodiscs (Fig. 8). Since CytbX is a computationally-derived sequence, with no natural homologues, this protein should have no intrinsic functional preference toward a specific membrane environment. Indeed, functionality was not explicitly considered during the original CytbX design process [16]. We would thus not expect that electron transport is necessarily enhanced by the presence of natural membrane lipids.

Interprotein electron transfer is complex, but the rate of such reactions are mainly a function of intercofactor distance (which can be >15 Å for protein cofactors), the reorganisation energy (λ), the intervening medium between the proteins, and the difference in redox potential between the two centres [39,40]. We show here that the redox potentials of CytbX vary only slightly between the micelle and nanodisc systems (Fig. 7), providing an equivalent driving force for electron transfer. We suggest that the slower rate of heme reduction in nanodiscs occurs because of the occlusion or deeper burial of CytbX within the membrane of the nanodisc, restricting the approach of water-soluble FLDR to the CytbX hemes. The reaction rate may also be affected by other factors such as differences in particle size [29], the immediate solvent environment surrounding CytbX (purified detergent *versus* crowded memtein), subtle changes in the CytbX fold, or charge repulsion of FLDR by the nanodisc polymer. Clearly, the specifics of this remain to be confirmed.

In summary, our results confirm prior observations of the *de novo* cytochrome CytbX and validate polymer nanodiscs as a new surfactant-free approach for working with this protein *in vitro*. Many properties of micelle-solubilised CytbX are preserved in these nanodisc preparations, consistent with *in situ* heme loading and suggesting that phenomena such as redox splitting may occur in cellular membranes. These robust CytbX nanodiscs will be a useful tool to enable our long-term goals of integrating CytbX with natural and artificial enzymes in bioengineering for catalysis, biomolecular circuitry, protocell fabrication, and synthetic bioenergetics.

## Supplementary Material

Supplementary data to this article can be found online at https://doi.org/10.1016/j.bbamem.2024.184392.

Supplementary data
