# Supplementary material for: Polymer nanodiscs support the functional extraction of an artificial transmembrane cytochrome
Source: Biochim Biophys Acta Biomembr. Author manuscript; Available in PMC 2025 Nov 18. (PMC7618366; doi:10.1016/j.bbamem.2024.184392)
Supplement: Supplementary data [file EMS208799-supplement-Supplementary_data.pdf]

SUPPLEMENTARY MATERIAL

Purification and characterisation of an artificial transmembrane cytochrome in polymer nanodiscs

Benjamin J Hardy, Holly C Ford, May Rudin, JL Ross Anderson and Paul Curnow*

School of Biochemistry, University of Bristol, UK
*Corresponding author. Email: p.curnow@bristol.ac.uk

The sequences of the synthetic genes used in this study are as follows.

>CytbX-GFP-His_gene
ATGGGCTCTCCTATTCTGCGCATCATTCACCTGATTTTGGCCTTGCTGGTTCTGATTACCGGACTTATCATGCTG
CTGAATACGTCAAATAGCCCCTATCTTCGCCTCATTCATTTTTTACTGGCACTGCTCGTGCTGATTACCGGTTGG
CTGATGCTAAAAAACGGTAGTAAGAGTCCGAGCCCGATCCTCCGTTTAATCCACATAATTCTGGCAATACTGG
TATTTATTACTGGCATCATTATGTTACTGAACACATCGAACAGCCCATTCCTGCGGATTTTGCATTTCATCCTTG
CGTTATTGGTCTTTATCACGGGCTTCCTTATGCTGAACCAGGCGGCCGCAGGTAAACCGATCCCGAATCCACTG
TTAGGGCTGGATTCCACCCTCGAGCTGGTGCCGCGCGGCAGCAGTAAAGGAGAAGAACTTTTCACTGGAGTT
GTCCCAATTCTTGTTGAATTAGATGGTGATGTTAATGGGCACAAATTTTCTGTCCGTGGAGAGGGTGAAGGTG
ATGCTACAAACGGAAAACTCACCCTTAAATTTATTTGCACTACTGGAAAACTACCTGTTCCGTGGCCAACACTT
GTCACTACTCTGACCTATGGTGTTCAATGCTTTTCCCGTTATCCGGATCACATGAAACGGCATGACTTTTTCAAG
AGTGCCATGCCCGAAGGTTATGTACAGGAACGCACTATATCTTTCAAAGATGACGGGACCTACAAGACGCGT
GCTGAAGTCAAGTTTGAAGGTGATACCCTTGTTAATCGTATCGAGTTAAAGGGTATTGATTTTAAAGAAGATG
GAAACATTCTTGGACACAAACTGGAGTACAACTTTAACTCACACAATGTATACATCACGGCAGACAAACAAAA
GAATGGAATCAAAGCTAACTTCAAAATTCGCCACAACGTTGAAGATGGTTCCGTTCAACTAGCAGACCATTAT
CAACAAAATACTCCAATTGGCGATGGCCCTGTCCTTTTACCAGACAACCATTACCTGTCGACACAATCTGTCCTT
TCGAAAGATCCCAACGAAAAGCGTGACCACATGGTCCTTCTTGAGTTTGTAACTGCTGCTGGGATTACACATG
GCATGGATGAGCTCTACAAACTCGAACACCACCACCACCACCACCACCACCACCAC**TGA**

Cloned between the NcoI/XhoI sites of pET28a(+)

>CytbX-GFP-His_protein
MGSPILRIIHLILALLVLITGLIMLLNTSNSPYLRLIHFLLALLVLITGWLMLKNGSKSPSPILRLIHIILAILVFITGIIMLLNT
SNSPFLRILHFILALLVFITGFLMLNQAAAGKPIPNPLLGLDSTLELVPRGSSKGEELFTGVVPILVELDGDVNGHKFSV
RGEGEGDATNGKLTLKFICTTGKLPVPWPTLVTTLTYGVQCFSRYPDHMKRHDFFKSAMPEGYVQERTISFKDDGT
YKTRAEVKFEGDTLVNRIELKGIDFKEDGNILGHKLEYNFNSHNVYITADKQKNGIKANFKIRHNVEDGSVQLADHY
QQNTPIGDGPVLLPDNHYLSTQSVLSKDPNEKRDHMVLLEFVTAAGITHGMDELYKLEHHHHHHHHHH-

>CytbX-His_gene
ATGGGCTCTCCTATTCTGCGCATCATTCACCTGATTTTGGCCTTGCTGGTTCTGATTACCGGACTTATCATGCTGCT
GAATACGTCAAATAGCCCCTATCTTCGCCTCATTCATTTTTTACTGGCACTGCTCGTGCTGATTACCGGTTGGCTGA
TGCTAAAAAACGGTAGTAAGAGTCCGAGCCCGATCCTCCGTTTAATCCACATAATTCTGGCAATACTGGTATTTAT
TACTGGCATCATTATGTTACTGAACACATCGAACAGCCCATTCCTGCGGATTTTGCATTTCATCCTTGCGTTATTGG
TCTTTATCACGGGCTTCCTTATGCTGAACCAGGCGGCCGCAGGTAAACCGATCCCGAATCCACTGTTAGGGCTGG
ATTCCACCCATCACCACCATCACCATCACCATCATCAT**TGA**

Cloned between the NcoI/XhoI sites of pET28a(+)
>CytbX-His_protein

MGSPILRIIHLILALLVLITGLIMLLNTSNSPYLRLIHFLLALLVLITGWLMLKNGSKSPSPILRLIHIILAILVFITGIIMLLNT
SNSPFLRILHFILALLVFITGFLMLNQAAAGKPIPNPLLGLDSTHHHHHHHHHH*

>CytbX-Strep_gene
ATGGGCTCTCCTATTCTGCGCATCATTCACCTGATTTTGGCCTTGCTGGTTCTGATTACCGGACTTATCATGCTGCT
GAATACGTCAAATAGCCCCTATCTTCGCCTCATTCATTTTTTACTGGCACTGCTCGTGCTGATTACCGGTTGGCTGA
TGCTAAAAAACGGTAGTAAGAGTCCGAGCCCGATCCTCCGTTTAATCCACATAATTCTGGCAATACTGGTATTTAT
TACTGGCATCATTATGTTACTGAACACATCGAACAGCCCATTCCTGCGGATTTTGCATTTCATCCTTGCGTTATTGG
TCTTTATCACGGGCTTCCTTATGCTGAACCAGGCGGCCGCACTGGTTCCGCGTGGATCCGGTGGTGGGTCTGGT
GGTGGGAGCGGTGGAGGCAGCTGGTCGCATCCGCAGTTTGAGAAGGGCGGCGGATCAGGCGGCGGATCCGG
CGGTGGCTCGTGGTCCCATCCGCAATTCGAGAAGGGTGGCGGCAGTGGTGGCGGCTCTGGCGGTGGGTCGTG
GAGCCACCCACAGTTCGAAAAGTGA

Cloned between the NdeI/XhoI sites of pET-29b(+).

>CytbX-Strep_protein
MGSPILRIIHLILALLVLITGLIMLLNTSNSPYLRLIHFLLALLVLITGWLMLKNGSKSPSPILRLIHIILAILVFITGIIMLLNTS
NSPFLRILHFILALLVFITGFLMLNQAAALVPRGSGGGSGGGSGGGSWSHPQFEKGGGSGGGSGGGSWSHPQFEK
GGGSGGGSGGGSWSHPQFEK-

FIGURE S1

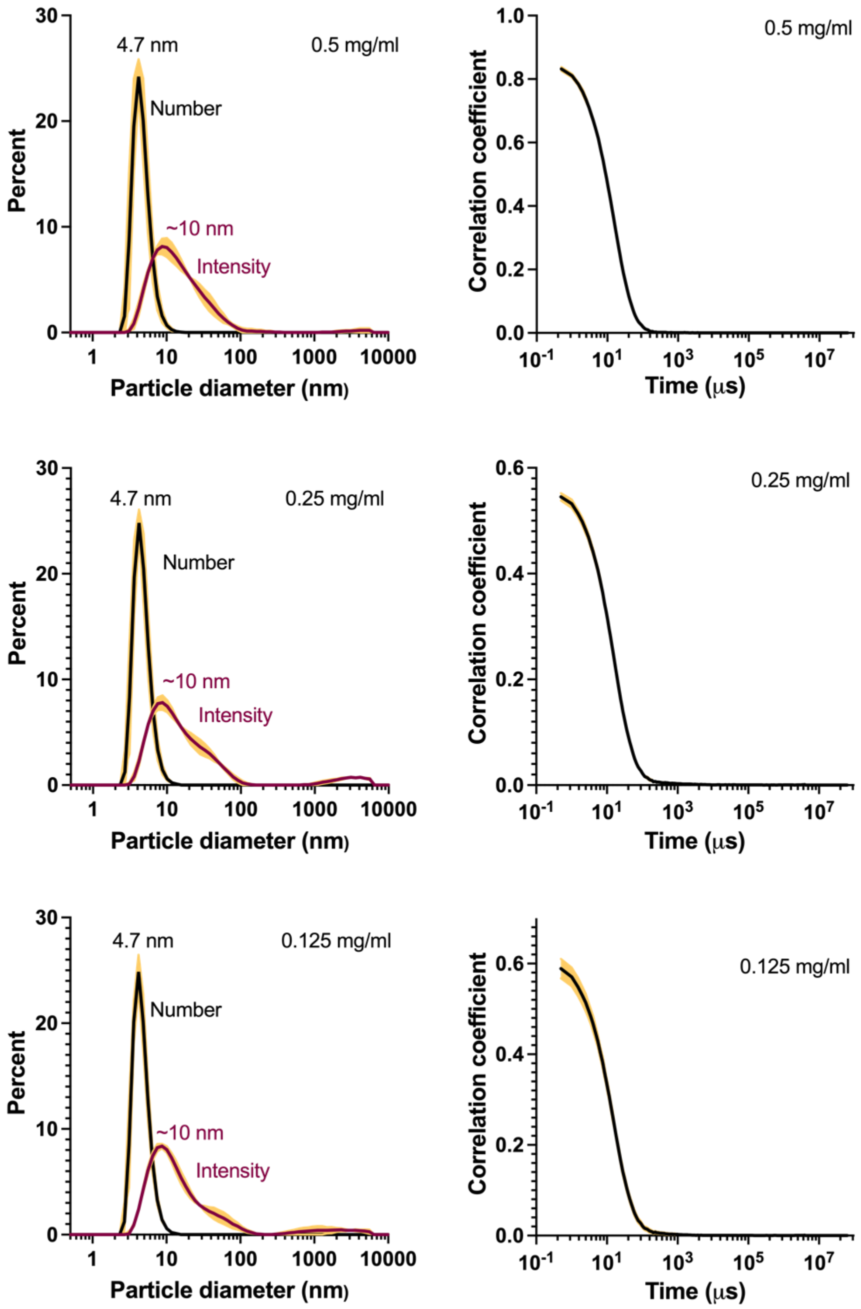

Figure S1. Dynamic Light Scattering data from CytbX in CyclAPol nanodiscs at different protein concentrations. Correlograms for each dataset are shown in the right-hand panels. All data are mean ± SD from three technical replicates, with errors bars in yellow. Data collected on Malvern Zetasizer Nano ZSP instrument.

FIGURE S2

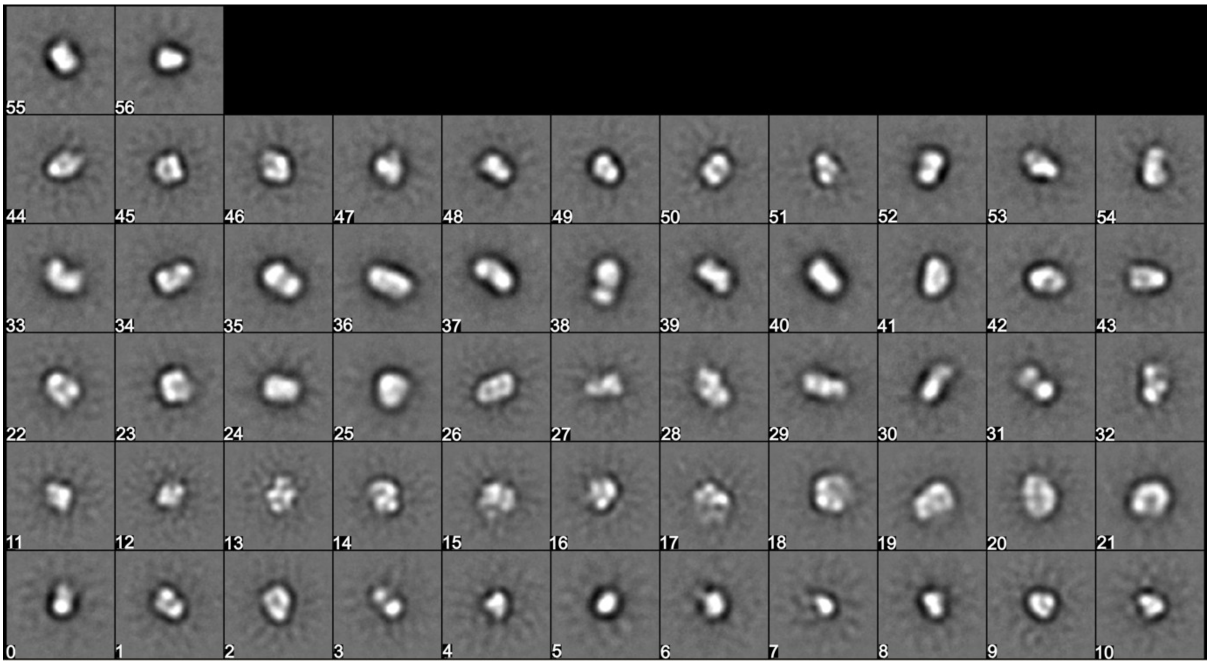

Figure S2. Full set of 2D class averages from negative stain electron micrographs of CytbX in CyclAPol nanodiscs, presented using the ‘Grid Display’ feature of EMAN2. The individual images of classes 2, 9, 21 and 45 are featured as representative examples in the main text.
